# Association between falls and dementia risk: Evidence from three cohort studies

**DOI:** 10.1515/jtim-2025-0057

**Published:** 2025-12-05

**Authors:** Aonan Li, Shaojiong Zhou, Jie Chang, Tao Wei, Bo Zhao, Yiwei Zhao, Zhibin Wang, Yi Xing, Yi Tang

**Affiliations:** Department of Neurology & Innovation Center for Neurological Disorders, Xuanwu Hospital, Capital Medical University, National Center for Neurological Disorders, Beijing, China; Neurodegenerative Laboratory of Ministry of Education of the Peoples Republic of China, Beijing, China; National Center for Neurological Disorders, Xuanwu Hospital, Capital Medical University, Beijing, China

**Keywords:** dementia, falls, polygenic risk scores, UK Biobank, China Health and Retirement Longitudinal Study

## Abstract

**Background and Objectives:**

The relationship between falls and dementia risk remains unclear. This study aims to investigate this association and assess the impact of genetic risk on this relationship.

**Methods:**

We included 265,416, 5,506, and 948 participants from the United Kingdom (UK) Biobank, China Health and Retirement Longitudinal Study (CHARLS), and Xuanwu cognitive cohort. A Cox model and logistic regression analysis were used to estimate the relationship between falls and dementia risk.

**Results:**

Falls showed significant associations with an elevated risk of dementia in both UK Biobank (single falls: Hazard Ratio [HR] 1.22, 95% Confidence Interval [CI]: 1.13-1.31; multiple falls: HR 1.73, 95% CI: 1.58-1.89), CHARLS cohorts (falls: HR 1.29, 95% CI: 1.11-1.50) and Xuanwu cognitive cohort (single falls: OR 1.72, 95% CI: 1.18-2.50; multiple falls: OR 1.89, 95% CI: 1.17-3.02). In addition, the results showed that falls were associated with cognitive decline during follow-up. Those who experienced single falls demonstrated reduced whole-brain gray matter volume (β = −1276.031, *P* = 0.029) and parahippocampal gyrus volume (β_left_ = −22.410, *P* = 0.011; β_right_ = −31.570, *P* = 0.001), whereas those experienced multiple falls, in addition to these findings, demonstrated increased deep white matter hyperintensities (β = 123.146, *P* = 0.004) and decreased gray matter volume in the hippocampus (β_left_ = −18.440, *P* = 0.002; β_right_ = −20.599, *P* = 0.001) and parahippocampal gyrus volume (β_left_ = −48.805, *P* < 0.001; β_right_ = −58.263, *P* < 0.001). Furthermore, falls and polygenic risk scores (PRSs) showed a significant interaction (*P* = 0.001), with higher dementia risk observed in individuals with multiple falls and high genetic risk.

**Conclusions:**

Falls are an early sign of dementia risk, and genetic susceptibility facilitates this association. Falls indicate potential atrophy in cognition-related brain regions. Falls are a simple and easily observable sign for dementia screening.

## Introduction

The annual incidence of falls in individuals aged 65 and older is as high as 35% and increases with age,^[1,2]^ particularly in the population of those with dementia.^[3,4]^ Falling may serve as a potential sign of dementia onset. However, the relevance of falls in cognitively healthy elderly individuals regarding the risk of dementia is underappreciated, owing to a lack of longitudinal studies following dementia outcomes among cognitively healthy elderly individuals after falls, as well as inconsistent conclusions on the topic in the literature. Several studies have suggested that falls may occur during the pre-dementia stage and are associated with subsequent cognitive impairment and risk of dementia.^[5, 6, 7]^ A recent cohort study suggested that the incidence of falls may be associated with the risk of preclinical Alzheimer’s disease (AD), preceding detectable cognitive changes.^[8]^ However, Jayakody *et al*. indicated that 1 year after falls, the risk of motoric cognitive risk syndrome was increased, whereas the risk of dementia was not.^[6]^ Hence, the precise association between falling and the risk of dementia remains unclear. Large-scale longitudinal follow-up studies are needed to elucidate the association between falls and the development of dementia.

Falls in elderly individuals may be an externalized phenotype of subclinical cognitive decline and brain structural atrophy in the predementia stages. Research indicates that differences in brain structures related to falls, such as the hippocampus, may explain why individuals with cognitive impairment and dementia are at increased risk of falls.^[9]^ Therefore, we speculate that falling is an observable indicator or manifestation of cerebral atrophy and may suggest a high risk of dementia. However, whether falls can alter brain structures to increase future dementia risk remains unknown. Furthermore, certain genotypes have been linked to the association between falls and dementia, with older adults who carry the apolipoprotein E (APOE) ε4 allele having an increased risk of dementia after falls.^[7,10]^ The onset of AD involves numerous pathogenic and risk genes, and the mechanisms are complex.^[11,12]^ Nevertheless, the role of genetic susceptibility in the association between falls and dementia risk is largely unknown. Polygenic risk score (PRS) is a powerful tool for comprehensively evaluating genetic susceptibility to diseases and stratifying risk.^[13]^ Hence, employing PRS is essential for understanding the effect of genetic susceptibility on the association between falls and dementia risk.

To support dementia screening and prevention, we studied the association between falls and dementia risk as well as the underlying mechanisms based on three cohorts from Europe and Asia. We found that falls serve as an early warning signal of cognitive decline in older adults that should prompt dementia screening, and that falls indicate potential structural changes in cognition-related brain regions. Using PRS to comprehensively verify the role of genetic susceptibility in this context, we also determine that genetic susceptibility facilitates the association between falls and the risk of dementia.

## Material and methods

### Study design and population

Data from three cohort studies were sourced from the UK Biobank, the CHARLS, and the Xuanwu cognitive cohort. The UK Biobank is a large-scale prospective cohort study that recruited approximately 500,000 volunteers aged 37-73 years from 2006 to 2010.^[14,15]^ Baseline interviews were conducted in 2006, with a median follow-up period of 13.7 years. All participants underwent baseline assessment at one of 22 evaluation centers located in England, Scotland, or Wales. Written informed consent was obtained from all participants, and ethical approval was granted by the National Health Service (NHS) North West Centre for Research Ethics Committee (ref: 16/NW/0274). The inclusion criteria for this study were: (1) individuals with a complete falls assessment and (2) individuals without cognitive impairment at baseline. The exclusion criteria were: (1) individuals diagnosed with dementia at baseline or within two years after baseline to minimize potential reverse causation, (2) individuals diagnosed with dementia before age 65, (3) individuals under the age of 65 at the last follow-up, and (4) individuals with fractures or other severe fall-related injuries requiring treatment. The study population was restricted to individuals showing a predisposition for late-onset dementia at baseline.

To further validate, we included patients from the CHARLS and Xuanwu cognitive cohorts. The inclusion and exclusion criteria for participants were consistent with the UK Biobank cohort. CHARLS is a comprehensive survey project aimed at collecting data on older adults.^[16]^ Participants aged 45 and above were recruited from 450 urban and rural areas across 30 provinces in China. Baseline interviews were conducted in 2011, with follow-up assessments in 2013, 2015, and 2018. The CHARLS study was approved by the Ethics Committee of Peking University, and all participants provided informed consent. The Xuanwu cognitive cohort consisted of all patients who were invited to participate while hospitalized in the Neurology Department of Xuanwu Hospital from 2021 to 2025. This case-control study was a comprehensive screening through telephone recall, with a total of 1,263 participants involved in the survey. Among the participants, 605 were diagnosed with dementia by doctors, and the controls were randomly selected from a non-dementia population to minimize bias. To optimize the balance of baseline characteristics between the dementia and control groups, a 1:1 propensity score matching (PSM) was performed based on age, sex, education level, and other factors. Ultimately, 948 participants (474 with dementia and 474 without dementia) were included in this study. This study was approved by the Ethics Committee of Xuanwu Hospital, Capital Medical University (No. 2024347).

### Falls

In the UK Biobank, falls data were collected at baseline and measured with an item that asked participants the following question: “Have you had any falls in the past year?” Participants could respond with options including “non-fall”, “single falls”, or “multiple falls.” Additionally, owing to limited reporting, individuals who fell and subsequently experienced fractures were not included in the analysis.

In the CHARLS study, falls data were collected at baseline and treated as a binary variable, with participants asked, “Have you experienced falls in the past two years?” Those who reported “yes” were classified as “falls”, while those who did not were classified as “non-fall.” Participants were asked, “How many falls required medical treatment in the past two years?” Those who had severe consequences of falls requiring treatment were excluded from the final analysis.

In the Xuanwu cognitive cohort, participants were contacted *via* telephone to inquire about their fall history and frequency before the dementia diagnosis. Responses were categorized as “non-fall”, “single falls”, or “multiple falls”, based on whether they reported no falls, one fall, or two or more falls, respectively. Participants who had fractures or other severe consequences of falls requiring treatment were also excluded from the analysis.

### Dementia

In UK Biobank, all-cause dementia was the primary study outcome, with secondary outcomes encompassing AD, vascular dementia (VD), and frontotemporal dementia (FTD). And, the diagnosis of dementia relied on algorithm-defined outcomes (ADOs).^[17]^ This approach was adopted by several other studies,^[18,19]^ and its reliability and efficacy were validated in a large-scale investigation involving 17,000 individuals in England.^[20]^ ADOs integrate information from baseline assessments and hospitalization data, including self-reports of medical conditions, procedures, and medication usage data, as well as mortality registration information obtained from linked hospital data.

In CHARLS, dementia is defined as either self-reported or physician-diagnosed dementia. Possible dementia is defined as a combination of cognitive impairment and functional impairment. This definition aligns with the dementia definition in the Diagnostic and Statistical Manual of Mental Disorders (DSM-IV; details in Supplementary) and the International Classification of Diseases, 10th Edition (ICD-10), and has been validated through a national population survey.^[21]^ Respondents with cognitive impairment in two or more domains (defined as scoring 1.5 standard deviations or more below the mean in each educational group) are considered to have cognitive impairment.^[22,23]^ Functional status is measured by Activities of Daily Living (ADL), with difficulty performing any ADL task being defined as functional impairment. In Xuanwu cognitive cohort, dementia was diagnosed based on inpatient records. The cases were defined as those first diagnosed with dementia between June 1, 2021, and March 31, 2025, according to ICD-10 codes (F00, F01, F02, F03, F05, G30), with a diagnosis age of ≥65 years.

### PRS

The standard PRS for AD was obtained as described elsewhere.^[24]^ A PRS for AD and dementia was derived from AD data and included common genetic variants linked to AD and dementia. PRS for AD were acquired following methods detailed previously.^[25]^ Single-nucleotide polymorphisms (SNPs) were chosen from previous AD genome-wide association studies (GWAS). Alleles at each SNP were weighted by their association strength in the discovery GWAS, summed, and z-standardized to create a PRS for all participants in the UK Biobank. The PRS for dementia was stratified into quintiles—"low" (quintile 1), "intermediate" (quintiles 2–4), and "high" (quintile 5)— with higher scores indicating a higher risk of dementia.

### Cognitive function assessment

At baseline enrollment and the initial brain imaging assessment, professional cognitive assessors administered cognitive function tests to all individuals using a computer touchscreen interface during UK Biobank assessment center visits.^[26]^ We specifically focused on changes in two cognitive domains: numeric memory (Numeric Memory Test) and abstract reasoning tasks (Fluid Intelligence). Detailed descriptions of cognitive functions are presented in Supplementary Table S11.

Cognitive function (episodic memory and executive function) tests are used to assess participants’ cognitive change in the CHARLS cohort. Consistent with previous studies,^[27,28]^ the episodic memory test includes immediate recall and delayed recall (10 unrelated words). The episodic memory score was calculated as the mean of two recall tasks (ranging from 0 to 10). The executive function score consisted of three subtests: time orientation (0-4), numerical ability (0-5), and pentagon drawing test (0-1), with a total score range from 0 to 10.

### Brain structure

The brain imaging data of UK Biobank included in our study were collected during the first follow-up in 2014. Magnetic resonance imaging (MRI) data were acquired using a standard Siemens Skyra 3T scanner. Based on previous studies, we evaluated the association between falls and the following brain metrics: total gray matter volume, deep white matter hyperintensities volume, hippocampal gray matter volume, and parahippocampal gray matter volume.^[29, 30, 31, 32]^

### Covariates

In UK Biobank, covariates were selected for inclusion based on previous literature reports and availability at baseline. At baseline, demographic data (age, sex, ethnicity, education, and body weight) were collected. Body mass index (BMI) was calculated by trained nurses and classified into three categories according to World Health Organization standards: underweight (<18.5 kg/m^2^), normal weight (18.5 to <25 kg/m^2^), overweight (25 to <30 kg/m^2^), and obesity (≥30 kg/m^2^). Scores on the Townsend deprivation index (TDI),^[33]^ derived from census data related to unemployment, vehicle ownership, household overcrowding, and occupation, were divided into high (<−2.08), medium (−2.08 to 1.40), and low (>1.40) levels. We also examined the occurrence of long-term morbidities, encompassing 42 different major chronic diseases. These conditions were assessed through verbal interviews conducted by medical professionals at assessment centers. The development of the long-term morbidities index originated from a cross-sectional study in Scotland and was later adapted for the UK Biobank.^[34]^ The inclusion of the number of long-term morbidities was motivated by its association with falls and lifespan.^[35, 36, 37]^ Detailed definitions of morbidities are presented in Supplementary Table S12.

In the CHARLS and Xuanwu cognitive cohort, covariates included demographic information, lifestyle, long-term morbidities and BMI, specifically age, sex (male/female), education (less than high school/high school or equivalent/ college and higher), smoking status (smoker/non-smoker), and drinking status (drinker/non-drinker). Additionally, household income was also included as a covariate in the CHARLS.

### Statistical analysis

In UK Biobank and CHARLS, the association of falls with dementia onset was quantified using Cox proportional hazard models, assessing HR and 95% CIs, leveraging follow-up duration as the temporal foundation. The proportional hazards assumption was evaluated visually through scaled Schoenfeld residuals. No evidence indicated that any variables in the analysis violated the proportional hazards assumption. Based on the Xuanwu cognitive cohort, we conducted a case-control study using logistic regression to estimate the odds ratio (OR) with a 95% CI between falls and incident dementia, adjusting for all covariates.

In UK Biobank time-to-event analysis, follow-up was initiated from baseline assessment and continued until the occurrence of the following events: first diagnosis of dementia, loss to follow-up, death, or last hospital admission date. The latest review dates are as follows: England, 31 October 2022; Scotland, 31 August 2022; and Wales, 31 May 2022. Events were considered based on the earliest occurrence.

We employed other sensitivity analysis to test the robustness of our model in UK Biobank. Specifically, to determine whether follow-up duration affected association, the primary analysis was segmented into three follow-up periods: ≤5 years, ≤10 years, and complete follow-up. In addition, we excluded individuals with movement disorders at baseline and further evaluated the relationship between falls and dementia risk.

In UK Biobank, to investigate differences in the association between falls and dementia risk across subgroups, we explored effect modification by sex, age, BMI, ethnicity, education, smoking status, and alcohol intake. Linear regression was used to analyze the maximum likelihood estimates of coefficients associated with falls and their relationship with brain structures and cognitive change. Furthermore, we employed a moderation model to assess whether PRS moderates the relationship between falls and brain structures.

All *P* values are based on two-tailed tests, with statistical significance defined as *P <* 0.05. Data cleaning and analysis were performed using Stata version 18 and R version 4.3.2 software.

## Results

A total of 265,416 individuals were included in the primary analysis in UK Biobank (Supplementary Figure S1). Over a median follow-up period of 13.7 years, 5415 participants received their first diagnosis of dementia. This comprised 2436 individuals diagnosed with AD, 1142 individuals diagnosed with VD, and 171 individuals diagnosed with FTD. At baseline, participants were aged between 50 and 73 years (mean age 60.3 [5.0] years), and 54.2% were female.

A total of 5506 individuals from the CHARLS were included in current study (Supplementary Figure S1). Of the participants, 2928 (53.2%) were female, and the baseline mean standard deviation (SD) age of 65.8 (6.2) years. After a median follow-up of 7 years, 813 participants were identified as dementia. In a case-control study of Xuanwu cognitive cohort, A total of 948 participants (474 with dementia and 474 without dementia) were included in analysis after PSM (Supplementary Figure S1). Participants in the dementia and control groups were perfectly matched, so the demographic characteristics (age, sex, education, smoking status, drinking status, BMI, and long-term morbidities) were similar in two groups (Standardized Mean Difference [SMD] <0.1). The baseline characteristics of the UK Biobank, CHARLS, and Xuanwu cognitive cohort studies participants are presented in Table 1, Supplementary Table S1, and Supplementary Table S2.

**Table 1:** Descriptive statistics for the full sample and by baseline falls and incident dementia in the UK Biobank

Item	Whole population	Falls at baseline			Incident dementia	
	Total (*N* = 265,416)	Non-fall (*N* = 211,825)	Single falls (*N* = 37,368)	Multiple falls (*N* = 16,223)	No (*N* = 260,001)	Yes (*N* = 5415)
Age, years, mean (SD)	60.3 (5.0)	60.2 (5.0)	60.7 (5.0)	60.6 (5.0)	60.2 (4.9)	64.8 (3.5)
Sex, *N* (%)						
female	143,978 (54.2)	108,969 (51.4)	24,541 (65.7)	10,468 (64.5)	141,410 (54.4)	2568 (47.4)
male	121,438 (45.8)	102,856 (48.6)	12,827 (34.3)	5755 (35.5)	118,591 (45.6)	2847 (52.6)
Ethnicity, *N* (%)						
White	244,527 (92.1)	195,509 (92.3)	34,272 (91.7)	14,746 (90.9)	239,525 (92.1)	5002 (92.4)
Mixed	8335 (3.1)	6575 (3.1)	1171 (3.1)	589 (3.6)	8143 (3.1)	192 (3.5)
Asian	8943 (3.4)	6911 (3.3)	1415 (3.8)	617 (3.8)	8799 (3.4)	144 (2.7)
Black	1126 (0.4)	900 (0.4)	151 (0.4)	75 (0.5)	1098 (0.4)	28 (0.5)
Other ethnic groups	670 (0.3)	556 (0.3)	75 (0.2)	39 (0.2)	661 (0.3)	9 (0.2)
Education level, *N* (%)						
CSEs	12,107 (4.6)	9510 (4.5)	1736 (4.6)	861 (5.3)	11,921 (4.6)	186 (3.4)
O-levels	70,241 (26.5)	55,480 (26.2)	10,143 (27.1)	4618 (28.5)	68,629 (26.4)	1612 (29.8)
A-levels	34,865 (13.1)	27,692 (13.1)	5098 (13.6)	2075 (12.8)	34,193 (13.2)	672 (12.4)
College or university degree	102,578 (38.6)	83,020 (39.2)	14,054 (37.6)	5504 (33.9)	100,829 (38.8)	1749 (32.2)
NVQ, HND, HNC, or equivalent	24,346 (9.2)	19,493 (9.2)	3207 (8.6)	1646 (10.1)	23,711 (9.1)	635 (11.7)
Other professional qualifications	21,279 (8.0)	16,630 (7.9)	3130 (8.4)	1519 (9.4)	20,718 (8.0)	561 (10.4)
Townsend Deprivation Index score, *N* (%)						
Lower	387,53 (14.6)	29,329 (13.8)	5963 (16.0)	3461 (21.3)	20,493 (7.9)	455 (8.4)
Middle	75,775 (28.5)	59,753 (28.2)	11,142 (29.8)	4880 (30.1)	97,309 (37.4)	2329 (43.0)
Higher	150,888 (56.8)	122,743 (57.9)	20,263 (54.2)	7882 (48.6)	142,199 (54.7)	2631 (48.6)
Smoking status, *N* (%)						
Never	144,830 (54.6)	116,048 (54.8)	20,454 (54.7)	8328 (51.3)	142,199 (54.7)	2631 (48.6)
Former	99,638 (37.5)	79,388 (37.5)	14,010 (37.5)	6240 (38.5)	97,309 (37.4)	2329 (43.0)
current	20,948 (7.9)	16,389 (7.7)	2904 (7.8)	1655 (10.2)	20,493 (7.9)	455 (8.4)
Alcohol consumption, *N* (%)						
Daily or most daily	64,967 (24.5)	52,751 (24.9)	8754 (23.4)	3462 (21.3)	63,626 (24.5)	1341 (24.8)
3 or 4 Times a week	65,180 (24.6)	53,074 (25.1)	8826 (23.6)	3280 (20.2)	64,084 (24.6)	1096 (20.2)
Once or twice a week	63,098 (23.8)	50,863 (24.0)	8657 (23.2)	3578 (22.1)	61,943 (23.8)	1155 (21.3)
1-3 Times a month	26,731 (10.1)	20,900 (9.9)	4023 (10.8)	1808 (11.1)	26,200 (10.1)	531 (9.8)
Special occasions only	27,288 (10.3)	20,577 (9.7)	4421 (11.8)	2290 (14.1)	26,587 (10.2)	701 (12.9)
Never or special occasions only	18,152 (6.8)	13,660 (6.4)	2687 (7.2)	1805 (11.1)	17,561 (6.8)	591 (10.9)
BMI, *N* (%)						
Underweight	1314 (0.5)	1061 (0.5)	171 (0.5)	82 (0.5)	1274 (0.5)	40 (0.7)
Normal weight	87,051 (32.8)	71,114 (33.6)	11,708 (31.3)	4229 (26.1)	85,316 (32.8)	1735 (32.0)
Overweight	115,631 (43.6)	93,444 (44.1	15,700 (42.0)	6487 (40.0)	113,265 (43.6)	2366 (43.7)
Obesity	61,420 (23.1)	46,206 (21.8)	9789 (26.2)	5425 (33.4)	60,146 (23.1)	1274 (23.5)
No. of long-term morbidities, N (%)						
None	90,731 (34.2)	75,954 (35.9)	11,369 (30.4)	3408 (21.0)	89,591 (34.5)	1140 (21.1)
1	87,205 (32.9)	70,718 (33.4)	11,958 (32.0)	4529 (27.9)	85,600 (32.9)	1605 (29.6)
2	50,044 (18.9)	38,959 (18.4)	7561 (20.2)	3524 (21.7)	48,767 (18.8)	1277 (23.6)
3	22,578 (8.5)	16,590 (7.8)	3773 (10.1)	2215 (13.7)	21,829 (8.4)	749 (13.8)
4	9012 (3.4)	6161 (2.9)	1588 (4.2)	1263 (7.8)	8657 (3.3)	355 (6.6)
≥5	5846 (2.2)	3443 (1.6)	1119 (3.0)	1284 (7.9)	5557 (2.1)	289 (5.3)
Genetic risk category, *N* (%)						
Low	51,769 (19.5)	41,349 (19.5)	7228 (19.3)	3192 (19.7)	51,220 (19.7)	549 (10.1)
Intermediate	15,5979 (58.8)	124,728 (58.9)	21,855 (58.5)	9396 (57.9)	153,529 (59.0)	2450 (45.2)
High	57,688 (21.7)	45,748 (21.6)	8285 (22.2)	3635 (22.4)	55,252 (21.3)	2416 (44.6)

BMI, body mass index; SD, standard deviation; CSEs, certificate of secondary education; NVQ, national vocational qualification; HND, higher national diploma; HNC, higher national certificate.

### Association between falls and dementia risk

**Figure 1:**
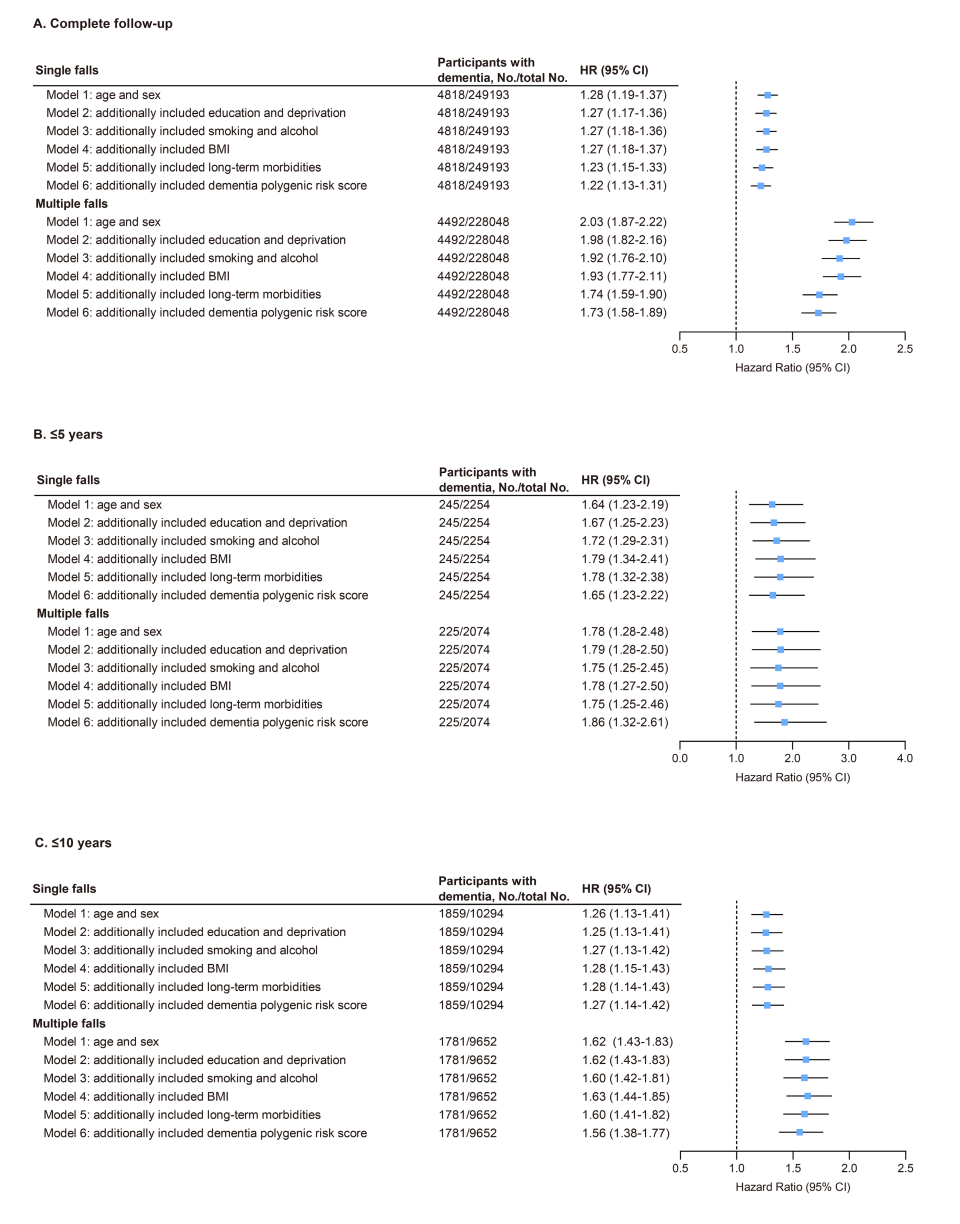
Association of falls with dementia risk in models with different sets of covariates and the fully adjusted model in UK Biobank. (A) Both single and multiple falls at baseline were associated with increased dementia risk after full adjustment (HR = 1.22 and 1.73, respectively; all *P* < 0.001). (B, C) Similar associations were observed in sensitivity analyses with follow-up limited to ≤5 years (single falls: HR = 1.65, *P* < 0.001; multiple falls: HR = 1.86, *P* < 0.001) and ≤10 years (single falls: HR = 1.27, *P* < 0.001; multiple falls: HR = 1.56, *P* < 0.001). Model 1, adjusted for age and sex. Model 2, included model 1 plus education and Townsend deprivation index. Model 3, included model 2 plus smoking status and alcohol intake. Model 4, included model 3 plus body mass index. Model 5, included model 4 plus long-term morbidities. Model 6, included model 5 plus dementia PRS. CI: confidence interval; HR, hazard ratio.

In UK Biobank, a significant positive association was found between falls and increased risk of incident dementia after adjusting for age and sex. Compared to individuals without a history of falls, individuals who experienced single or multiple falls at baseline had a higher risk of developing dementia over the entire follow-up period (single falls, HR = 1.28, 95% CI: 1.19-1.37; multiple falls, HR = 2.03, 95% CI: 1.87-2.22; Figure 1). After further adjustment for all confounding factors (age, sex, education level, TDI score, BMI, smoking, alcohol consumption, and presence of long-term morbidities), higher dementia risks remained statistically significant both in individuals experiencing a single fall and in individuals experiencing multiple falls compared to individuals who did not experience any fall (single falls: HR = 1.22, 95% CI: 1.13-1.31; multiple falls: HR = 1.73, 95% CI: 1.58-1.89; Figure 1).

In CHARLS, consistent with the results from the UK Biobank, individuals who experienced falls had a significantly higher risk of dementia compared to non-fall (HR = 1.45, 95% CI: 1.25-1.69), after adjusting for age and sex. After adjusting for all confounding factors, the results still showed a higher risk of dementia in falls (HR = 1.29, 95% CI: 1.11-1.50; Supplementary Table S3). In Xuanwu cognitive cohort, we examined the association between single and multiple falls and dementia risk. The results indicated that individuals who experienced single falls had a higher risk of dementia compared to non-fallers (OR = 1.72, 95% CI: 1.18-2.50). Further analysis revealed that individuals with multiple falls also had an increased risk of dementia (OR = 1.89, 95% CI: 1.17-3.02; Supplementary Table S4).

### Association among falls, brain structures, and cognitive changes

**Figure 2:**
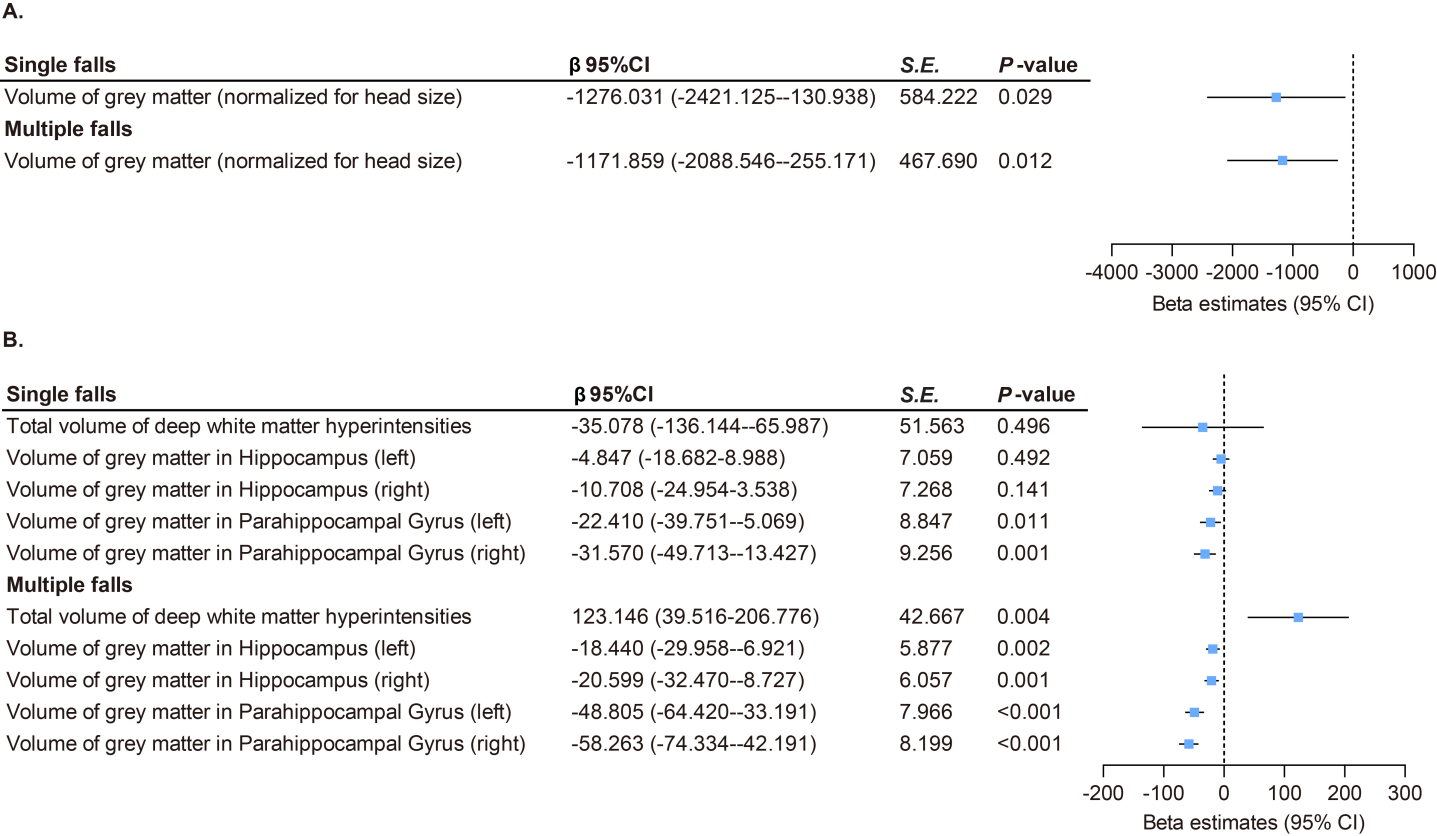
Association between falls and brain structures in UK Biobank. (A) Both single (β = –1276.031, *P* = 0.029) and multiple (β = –1171.859, *P* = 0.012) falls were linked to reduced total gray matter volume. (B) Both single and multiple falls were linked to reduced gray matter volumes of parahippocampal gyrus (β range: –58.263 to –22.410, all *P* < 0.05). Multiple falls were associated with increased white matter hyperintensities (β = 123.146, *P* = 0.004) and decreased gray matter volumes in hippocampus (β range: –20.599 to –18.440, all *P* < 0.05). P values were fully adjusted for age, sex, education, Townsend deprivation index, alcohol consumption, smoking status, BMI, and the number of long-term morbidities. CI: confidence interval; S. E., standard error.

In UK Biobank, brain structural analysis by MRI showed that individuals who experienced a single fall had reduced gray matter volume in some brain structures, mainly reflected in gray matter volume (β = −1276.031, *P* = 0.029), left parahippocampal gray matter volume (β = −22.410, *P* = 0.011), and right parahippocampal gray matter volume (β = −31.570, *P* = 0.001). Additionally, individuals with multiple falls had an increased volume in white matter hyperintensities (β = 123.146, *P* = 0.004) and decreased volumes in gray matter (β = −1,171.859, *P* = 0.012), left hippocampus gray matter (β = −18.440, *P* = 0.002), right hippocampus gray matter (β = −20.599, *P* = 0.001), left parahippocampal gyrus gray matter (β = −48.805, *P* < 0.001), and right parahippocampal gyrus gray matter (β = −58.263, *P* < 0.001; Figure 2).

We further analyzed the association between falls and cognitive changes based on the UK Biobank. Our analysis revealed that individuals who experienced falls also had cognitive decline. After adjusting for all factors, individuals who experienced a single fall exhibited cognitive decline in abstract reasoning (β = −0.064, *P* = 0.035) and numerical memory (β = −0.068, *P* = 0.017). Furthermore, those who experienced multiple falls exhibited cognitive decline both in abstract reasoning (β = −0.126, *P* < 0.001) and numerical memory (β = −0.099, *P* < 0.001) compared to individuals who did not experience falls (Supplementary Figure S5).

In CHARLS, we further analyzed the association between falls and cognitive changes. The results showed that the falls experienced cognitive decline in episodic memory (β = −0.029, *P* = 0.013), and also exhibited worse performance in executive function (β = −0.041, *P* < 0.001; Supplementary Table S5).

In addition, we examined the link between brain structures and cognitive changes in UK Biobank. After adjusting for all factors, our analysis revealed positive correlations between abstract reasoning and hippocampal gray matter volume (β_left_ = 0.119, *P <* 0.001; β_right_ = 0.114, *P* < 0.001) and parahippocampal gyrus gray matter volume (β_left =_ 0.117, *P* < 0.001; β_right_ = 0.111, *P* < 0.001). Numeric memory showed negative correlations with deep white matter hyperintensities volume (β = -0.035, *P* < 0.001) and positive correlations with hippocampal gray matter volume (β_left_ = 0.078, *P* < 0.001; β_right_ = 0.063, *P* = 0.002) and parahippocampal gyrus gray matter volume (β_left_ = 0.078, *P* < 0.001; β_right =_ 0.122, *P* < 0.001; Supplementary Table S6).

### Moderating effects of genetic risk on association between falls and dementia Risk

**Figure 3:**
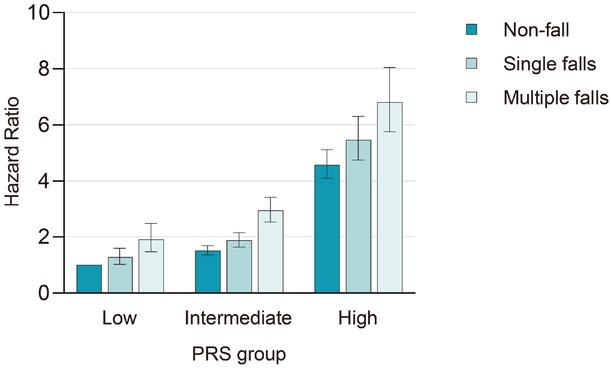
Risk of incident dementia disease according to genetic risk and falls in the UK Biobank. Dementia risk increased with both falls and PRS, with the highest risk in individuals experiencing multiple falls and high PRS (HR = 6.81, 95% CI: 5.77-8.05). P values were fully adjusted for age, sex, education, Townsend deprivation index, alcohol consumption, smoking status, BMI, and the number of long-term morbidities. PRS, polygenic risk score.

We found a positive link between dementia PRS tertiles and dementia risk (Supplementary Table S7). Furthermore, we investigated the combined impact of fall phenotype and PRS on dementia incidence, which showed that, compared to individuals without a history of falls with low PRS scores, those with multiple falls and high PRS scores exhibited the highest risk of dementia (HR = 6.81, 95% CI: 5.77-8.05; Figure 3; Table 2).

**Table 2:** Risk of incident dementia according to genetic risk and falls in the UK Biobank

Item	Low PRS	Intermediate PRS	High PRS
Non-fall	1 [reference]	1.51 (1.35-1.69)	4.58 (4.10-5.12)
Single falls	1.28 (1.02-1.60)	1.88 (1.63-2.16)	5.47 (4.76-6.30)
Multiple falls	1.91 (1.47-2.48)	2.94 (2.53-3.43)	6.81 (5.77-8.05)

Adjusted for age, sex, education, Townsend deprivation index, alcohol consumption, smoking status, body mass index, and the number of long-term morbidities. PRS, polygenic risk score.

To explore the interaction between falls and different PRSs, we conducted a stratified analysis. The result revealed a significant interaction between falls and PRS, indicating a gene-environment interaction in dementia risk (*P* value for interaction = 0.001; Supplementary Table S8).

### Moderating effects of genetic risk on association between falls and brain structures

We further evaluated the moderating effect of PRS on the association between falls and brain structures using a moderation model. No significant moderating effects of PRS were found on the association between falls and brain structures (including total grey matter volume, deep white matter hyperintensities volume, hippocampus grey matter volume, and parahippocampal gyrus grey matter volume) after adjusting for all covariates (All *P* > 0.05; Supplementary Table S9).

### Sensitivity analyses

To explore the robustness of the results, we conducted a sensitivity analysis based on the UK Biobank. Firstly, we excluded individuals with movement disorders at baseline. Further analysis revealed consistent results, showing that both single falls (HR = 1.23, 95% CI: 1.14-1.33) and multiple falls (HR = 1.66, 95% CI: 1.51-1.83) had a higher dementia risk compared to non-fallers after adjusting for all covariates (Supplementary Figure S6).

Subsequently, we detected consistent associations when the censoring time was limited to ≤5 years and ≤10 years. The fully adjusted risk ratios for single falls were 1.65 (95% CI: 1.23-2.22) and 1.27 (95% CI: 1.14-1.42), for ≤5 years and ≤10 years respectively, whereas, for multiple falls, the risk ratios were 1.86 (95% CI: 1.32-2.61) and 1.56 (95% CI: 1.38-1.77), respectively (Figure 1). Therefore, the association pattern remained unchanged compared to the primary analyses, underscoring the robustness of the findings.

### Subgroup analyses

To further explore the relationship between falls and dementia subtypes, we did further subgroup analyses in UK Biobank. We found that participants experiencing single and multiple falls were at higher risk of AD and VD, and in addition, we found that those experiencing multiple falls were associated with FTD risk, after adjusting for all confounding factors (Supplementary Figure S2-S4).

To further investigate the relationship between falls and dementia risk in different populations, we performed subgroup analyses based on UK Biobank. As shown in Table S10, individuals younger than 60 years of age exhibited a higher risk of developing dementia after falls than individuals older than 60 years of age (*P* = 0.002). A higher risk of developing dementia after falls was also observed in individuals with a history of smoking (*P* = 0.016). No significant effect change was observed when stratifying data by sex, BMI, ethnicity, education, or alcohol intake (Supplementary Table S10).

## Discussion

In this study encompassing two prospective cohorts and a case-control study, we examined the relationship between falls and dementia risk. The results confirmed a significant association between falls and dementia risk. Furthermore, analysis revealed additional intriguing findings in the UK Biobank. A positive association between falls and the risk of incident dementia, as well as its subtypes, including AD, VD, and FTD. Consistent results were obtained in a sensitivity analysis, with the direction and strength of this association remaining consistent over follow-up periods of 5 years and 10 years. Subgroup analyses revealed that falls may serve as a warning signal for increased risk of dementia, particularly in young individuals. Furthermore, our investigation indicated that individuals with a history of falls exhibited cognitive decline and cortical atrophy predominantly in the hippocampus and parahippocampal gyrus, as well as increased deep white matter hyperintensities volume compared to individuals without a history of falls. There was an interaction between falls and genetic predisposition in dementia events, with the highest dementia risk found in individuals with a history of multiple falls and high PRS. These robust results suggest that falls may be a critical indicator of dementia risk, providing family and primary care physicians a metric to detect individuals with a high risk of dementia without expensive equipment, highly trained personnel, or specialized facilities.

Falls are a simple and easily observable parameter suitable for universal screening in various populations and are closely associated with the integrity of the central nervous system. In the context of cognitive impairment, recurrent falls have been established as being related to deteriorating performance on cognitive assessments in older adults.^[38]^ However, few studies have studied the association between falls and dementia risk, and reliable conclusions are still lacking. A study conducted by Einstein Aging Research found that falls can predict declines in cognitive and motoric cognitive risk, but they are not associated directly with the risk of dementia.^[6]^ Another retrospective cohort study indicated that falls with severe comorbidities are associated with the risk of dementia 1 year later in the elderly.^[39]^ Long-term longitudinal follow-up studies in cognitively healthy individuals are needed to further clarify the causal relationship between falls and dementia risk. Therefore, our study further investigates the relationship between falls and dementia risk through long-term longitudinal cohort follow-up data, excluding individuals who developed cognitive impairment during the baseline and the first two years after baseline. We further expanded our previous study by examining the association between falls and cognitive changes and brain structures, as well as the role of genetic susceptibility in falls and the risk of dementia. Additionally, we conducted sensitivity and subgroup analyses, and we investigated associations between falls and risk of dementia and its subtypes, providing a more comprehensive explanation of these relationships using a large-scale longitudinal cohort.

Falls are associated with the risk of different types of dementia (AD, VD, FTD). Notably, a 12-month longitudinal study involving cognitively normal community-dwelling older adults found that falls were significantly associated with elevated levels of tau/Aβ42 and brain amyloid protein in cerebrospinal fluid. These biomarkers were validated in individuals with mild cognitive impairment and AD.^[40]^ White matter hyperintensities (WMH), as a significant radiological marker for VD, are closely associated with slower gait speed, balance impairment, and increased fall risk.^[41]^ Our findings further confirm that multiple falls are associated with an increased burden of WMH, suggesting that falls may represent an early clinical manifestation of subcortical white matter lesions. Additionally, previous studies have shown that falls and gait abnormalities are associated with abnormal tau protein levels.^[42,43]^ The pathology of FTD, such as abnormal tau protein, may begin years before clinical symptoms emerge and could affect subcortical structures involved in gait and balance control. Multiple falls may reflect one of the manifestations of this widespread and early pathological change. Consistent with previous findings,^[6,38]^ we found that falls are associated with later cognitive decline. It is unclear how repeated falls are associated with cognitive decline, but it may be due to shared pathophysiological pathways.^[44, 45, 46]^ For example, overlapping neural networks involved in both cognition and gait control may partly account for this association.^[9,47]^ These findings suggest that falls may serve as an early sign of future cognitive decline and may aid in the identification of populations at risk for cognitive impairment. In addition, early falls intervention may reduce the incidence of cognitive impairment.^[48]^ A study on fall-related traumatic brain injury confirmed that brain injuries caused by falls increase the risk of cognitive impairment.^[49]^ Moreover, exercise game interventions not only improve balance and prevent falls in the elderly but also enhance cognitive function. Therefore, we believe that fall prevention and intervention strategies may prevent traumatic brain injuries and, to some extent, reduce the risk of cognitive impairment and dementia.^[50]^

Neuropathologic changes in dementia can begin up to 20 years before diagnosis,^[51]^ and alterations in brain structures, such as hippocampal volume, may be linked to cognitive function.^[52]^ Studying these changes could help elucidate the association between falls and dementia risk. Our study showed that falls predict the presence of atrophy in cognition-related brain regions, which provides new insights into the underlying mechanisms linking falls to the risk of dementia. A previous observational cohort study of 77 healthy elderly individuals indicated a correlation between falls and larger hippocampal volume.^[53]^ However, our large-scale cohort study found that individuals with a history of falls exhibited smaller hippocampal gray matter volume than individuals without a history of falls. This inconsistency between studies may be attributed to factors such as insufficient sample size, differing age restrictions, type of study, and variations in follow-up duration. In our present study, building upon previous research, we identified a negative association between falls and overall brain gray matter volume as well as a positive correlation with white matter hyperintensities. These changes in brain structures may be foundational to the decline in cognitive abilities related to falls. We further found that individuals with a history of falls exhibited greater loss of gray matter in the overall cortex, hippocampus, and parahippocampal gyrus. These regional alterations were associated with early cognitive deficits in learning and memory, culminating in whole-brain atrophy.^[54,55]^ Furthermore, white matter hyperintensities are considered a critical indicator of cerebral vascular burden in the aging brain as well as a significant predictor of cognitive decline.^[56,57]^ Other studies have suggested that traumatic brain injury,^[49]^ fear,^[58]^ and fractures ^[42]^ following falls may increase the risk of dementia. The transient cerebral ischemia detected during a fall, such as cardiogenic syncope, can lead to cognitive decline.^[59]^ Our results not only confirm the findings of previous studies but also provide comprehensive evidence that falls are a screening indicator for dementia. However, the pathophysiological mechanisms underlying brain structural changes associated with dementia after falls need to be more thoroughly researched. This also serves as a reminder to clinicians to prioritize MRI scanning of individuals who have fallen, to rule out the adverse effects of changes in brain structures after falls.

Falls are key screening indicators for people at high genetic risk for dementia. Several studies indicated that the *APOE* ε4 allele can predict dementia risk after falls.^[7,49,60]^ Another study examined the risk of falls influenced by the *APOE* ε4 allele in 810 carriers of the allele.^[61]^ Previous research indicated that high genetic risk is associated with smaller hippocampal volume and white matter lesions or hyperintensities,^[62, 63, 64]^ which may contribute to increased dementia risk. In our present study, we used a more comprehensive index (dementia PRS) to investigate the effects of genetic determinants on the association between falls and dementia risk. We found that a higher risk of incident dementia was linked to falls and elevated dementia PRS. Notably, our analyses uncovered a significant interaction between PRS tertiles and falls, indicating a gene-environment interaction in dementia onset. Specifically, individuals with high PRS and incidence of falls had a 6.81 times higher risk of dementia compared to individuals with low PRS and incidence of falls. In all PRS tertiles, individuals who did not experience falls had a significantly reduced risk of dementia. Falls are correlated with biomarkers of neurodegenerative disease, including hippocampal volume,^[65]^ and genetic risk is tightly intertwined with pathways contributing to tau and β-amyloid aggregation, particularly in the hippocampus, and especially in early AD.^[66]^ Therefore, falls should be more intentionally monitored in individuals at high genetic risk of dementia.

Our findings suggest that falls may serve as a valuable indicator for dementia screening, with important clinical implications for dementia prevention and early intervention.^[48]^ Our findings also suggest that, when individuals present with frequent falls, they should be monitored for changes in cognitive function and dementia, particularly in young adults with better cognitive function, and early interventions should be developed to prevent cognitive decline.

### Strengths and limitations

Our study has several important strengths, including firsthand evidence from three cohorts representing different ethnicities, a large sample size, and long-term follow-up. These factors collectively enhance the credibility of our findings. In addition, we investigated the associations between falls and various types of dementia and detailed their relationships. Employing brain structures and PRS analysis, we investigated possible mechanisms through which falls may influence the risk of dementia. However, our study also has some limitations. First, falls may be a manifestation of other risk factors, such as environmental factors, gait disorders, and behavioral factors.^[67, 68, 69]^ Still, the literature has confirmed that the above factors are related to the risk of dementia.^[70,71]^ Therefore, dementia caused by these factors may manifest itself as falls. Second, dementia cases in UK Biobank and CHARLS were primarily identified through hospital records and self-reported diagnoses, which may have led to case under detection. However, prior studies indicate good agreement with primary care data,^[20]^ and self-reported history is generally reliable. Third, UK Biobank measured covariates like BMI, alcohol use, and morbidities only at baseline, preventing assessment of their temporal changes on outcomes. Further research is needed to address this limitation. Fourth, dementia subtypes in UK Biobank were classified using electronic health records rather than biomarkers or imaging, which may risk misclassification. Therefore, the association between falls and dementia subtypes requires further investigation. Fifth, the cross-sectional, self-reported nature of falls data may introduce recall bias and misclassification, potentially underestimating the true association.^[72]^ Although longitudinal designs mitigate this limitation, they remain susceptible to attrition bias. Despite robust sensitivity analyses, studies with longer follow-up and more comprehensive data are needed. Sixth, women comprised only half of dementia patients in the UK Biobank, lower than expected from epidemiological data.^[73,74]^ This may reflect differences in sampling, diagnosis, or screening, as well as sociocultural influences on healthcare-seeking, potentially introducing gender bias. Future research should examine gender-related differences in early dementia presentation. Seventh, despite adjustment for comorbidities such as cerebrovascular disease and Parkinson’s disease, shared risk factors between falls and dementia cannot be entirely excluded. Randomized controlled trials of fall interventions are needed to elucidate potential causal links. Eighth, although key covariates were adjusted for, residual differences between populations (*e.g*., living environment) may remain. Future studies using harmonized data across cohorts are needed to improve comparability.

## Conclusions

We found that falls increase the risk of incident dementia, and genetic susceptibility facilitates this association, highlighting falls as an easily monitored risk factor for dementia. Falls may serve as an early sign of cognitive decline and region-specific brain atrophy. Therefore, future interventions and preventive measures targeting falls may help reduce the incidence of dementia events.

## Supplementary Information

Supplementary materials are only available at the official site of the journal (www.intern-med.com).

## Supplementary Material

Supplementary Material Details
