# Supplementary material for: Association between falls and dementia risk: Evidence from three cohort studies
Source: J Transl Int Med. 2025 Dec 5;13(6):568–81. doi: 10.1515/jtim-2025-0057 (PMC12721366; doi:10.1515/jtim-2025-0057)
Supplement: Supplementary file 1 — Supplementary Material Details [file jtim-2025-0057_sm.pdf]

Supplementary Material

Supplementary Figure S1 Study flow charts for three cohorts

Supplementary Figure S2 Association of falls with Alzheimer's disease in models with different sets of covariates and the fully adjusted model in the UK Biobank

Supplementary Figure S3 Association of falls with vascular dementia in models with different sets of covariates and the fully adjusted model in the UK Biobank

Supplementary Figure S4 Association of falls with frontotemporal dementia in models with different sets of covariates and the fully adjusted model in the UK Biobank

Supplementary Figure S5 Association between falls and cognitive decline in the UK Biobank

Supplementary Figure S6 Association between falls and the risk of dementia excluding movement disorders in the UK Biobank

Supplementary Table S1 General characteristics of participants at baseline in the CHARLS

Supplementary Table S2 General characteristics of participants after propensity score matching in the Xuanwu cognitive cohort

Supplementary Table S3 Association between falls and incident dementia in the CHARLS

Supplementary Table S4 Association between falls and incident dementia in the Xuanwu cognitive cohort

Supplementary Table S5 Association between falls and cognitive decline in the CHARLS

Supplementary Table S6 Association between brain structures and cognitive change in the UK Biobank

Supplementary Table S7 Association between PRS and incident dementia in the UK Biobank

Supplementary Table S8 Risk of incident dementia according to genetic and falls in the UK Biobank

Supplementary Table S9 Moderating effects of genetic risk on association between falls and brain structures in the UK Biobank

Supplementary Table S10 Association between falls and the risk of dementia stratified by subgroups in the UK Biobank

Supplementary Table S11 Summary of cognitive functions assessed in the UK Biobank

Supplementary Table S12 Definition and list of long-term morbidities in the UK Biobank

Case definition of dementia in relation to DSM-IV criteria

This additional file has been provided by the authors to give readers additional information about their work.

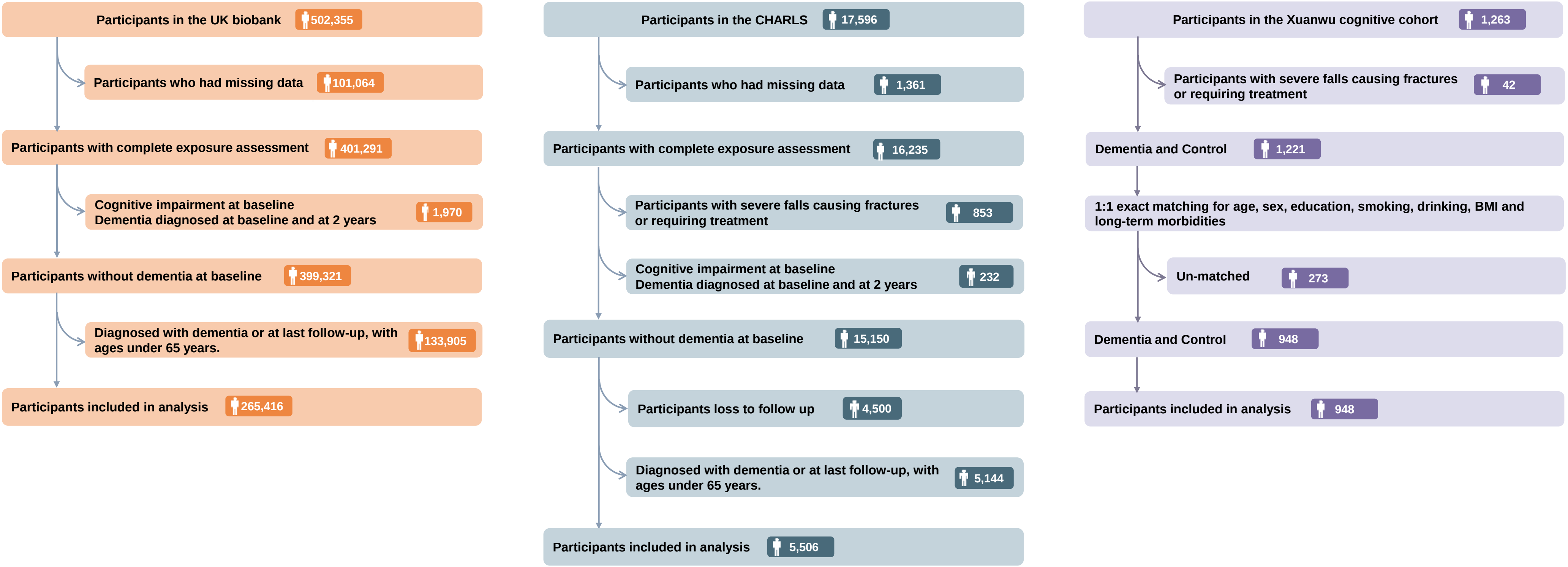

Supplementary Figure S1: Study flow charts for three cohorts. UK: United Kingdom; CHARLS: China Health and Retirement Longitudinal Study.

Single falls	Participants with AD, No./total No.	HR (95% CI)
Model 1: age and sex	2197/249193	1.20 (1.09-1.35)
Model 2: additionally included education and deprivation	2197/249193	1.19 (1.08-1.33)
Model 3: additionally included smoking and alcohol	2197/249193	1.19 (1.07-1.34)
Model 4: additionally included BMI	2197/249193	1.21 (1.08-1.34)
Model 5: additionally included long-term morbidities	2197/249193	1.19 (1.06-1.32)
Model 6: additionally included dementia polygenic risk score	2197/249193	1.17 (1.05-1.30)
**Multiple falls**		
Model 1: age and sex	2036/228048	1.71 (1.54-2.01)
Model 2: additionally included education and deprivation	2036/228048	1.71 (1.49-1.95)
Model 3: additionally included smoking and alcohol	2036/228048	1.67 (1.46-1.91)
Model 4: additionally included BMI	2036/228048	1.71 (1.49-1.96)
Model 5: additionally included long-term morbidities	2036/228048	1.61 (1.40-1.84)
Model 6: additionally included dementia polygenic risk score	2036/228048	1.58 (1.38-1.81)

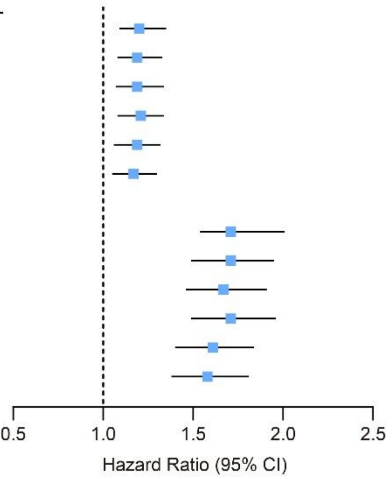

Supplementary Figure S2: Association of falls with Alzheimer's disease in models with different sets of covariates and the fully adjusted model in the UK Biobank. Both single and multiple falls at baseline were associated with increased Alzheimer's disease risk after full adjustment (HR = 1.17 and 1.58, respectively; all $P < 0.001$). Model 1, adjusted for age and sex. Model 2, included model 1 plus education and Townsend deprivation index. Model 3, included model 2 plus smoking status and alcohol intake. Model 4, included model 3 plus body mass index. Model 5, included model 4 plus long-term morbidities. Model 6, included model 5 plus dementia polygenic risk score. UK: United Kingdom; CI: confidence interval; HR: hazard ratio.

Single falls	Participants with VD, No./total No.	HR (95% CI)
Model 1: age and sex	988/249193	1.55 (1.33-1.81)
Model 2: additionally included education and deprivation	988/249193	1.53 (1.32-1.79)
Model 3: additionally included smoking and alcohol	988/249193	1.54 (1.32-1.79)
Model 4: additionally included BMI	988/249193	1.52 (1.31-1.77)
Model 5: additionally included long-term morbidities	988/249193	1.44 (1.24-1.68)
Model 6: additionally included dementia polygenic risk score	988/249193	1.43 (1.23-1.67)
**Multiple falls**		
Model 1: age and sex	926/228048	2.73 (2.29-3.25)
Model 2: additionally included education and deprivation	926/228048	2.64 (2.22-3.15)
Model 3: additionally included smoking and alcohol	926/228048	2.53 (2.12-3.02)
Model 4: additionally included BMI	926/228048	2.46 (2.07-2.93)
Model 5: additionally included long-term morbidities	926/228048	2.06 (1.72-2.46)
Model 6: additionally included dementia polygenic risk score	926/228048	2.05 (1.72-2.46)

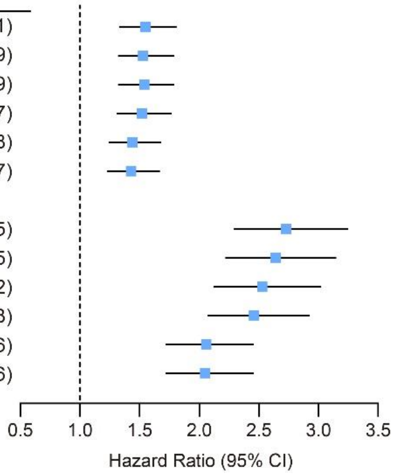

Supplementary Figure S3: Association of falls with vascular dementia in models with different sets of covariates and the fully adjusted model in the UK Biobank. Both single and multiple falls at baseline were associated with increased vascular dementia risk after full adjustment (HR = 1.43 and 2.05, respectively; all $P < 0.001$). Model 1, adjusted for age and sex. Model 2, included model 1 plus education and Townsend deprivation index. Model 3, included model 2 plus smoking status and alcohol intake. Model 4, included model 3 plus body mass index. Model 5, included model 4 plus long-term morbidities. Model 6, included model 5 plus dementia polygenic risk score. UK: United Kingdom; CI: confidence interval; HR: hazard ratio; BMI: body mass index; VD: vascular dementia.

Single falls	Participants with FTD, No./total No.	HR (95% CI)
Model 1: age and sex	151/249193	1.14 (0.74-1.75)
Model 2: additionally included education and deprivation	151/249193	1.13 (0.73-1.74)
Model 3: additionally included smoking and alcohol	151/249193	1.13 (0.73-1.73)
Model 4: additionally included BMI	151/249193	1.13 (0.73-1.75)
Model 5: additionally included long-term morbidities	151/249193	1.12 (0.73-1.73)
Model 6: additionally included dementia polygenic risk score	151/249193	1.12 (0.72-1.73)
**Multiple falls**		
Model 1: age and sex	146/228048	2.19 (1.36-3.51)
Model 2: additionally included education and deprivation	146/228048	2.13 (1.32-3.42)
Model 3: additionally included smoking and alcohol	146/228048	2.06 (1.28-3.32)
Model 4: additionally included BMI	146/228048	2.08 (1.29-3.36)
Model 5: additionally included long-term morbidities	146/228048	2.06 (1.27-3.33)
Model 6: additionally included dementia polygenic risk score	146/228048	2.04 (1.26-3.32)

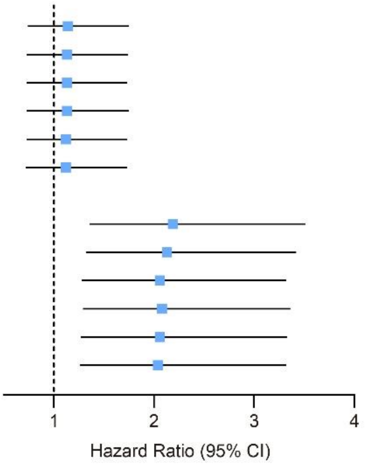

Supplementary Figure S4: Association of falls with frontotemporal dementia in models with different sets of covariates and the fully adjusted model in the UK Biobank. Multiple falls at baseline were associated with increased frontotemporal dementia risk after full adjustment (HR = 2.04; $P < 0.001$). Model 1, adjusted for age and sex. Model 2, included model 1 plus education and Townsend deprivation index. Model 3, included model 2 plus smoking status and alcohol intake. Model 4, included model 3 plus body mass index. Model 5, included model 4 plus long-term morbidities. Model 6, included model 5 plus dementia polygenic risk score. UK: United Kingdom; CI: confidence interval; HR: hazard ratio; BMI: body mass index.

Single falls	β 95%CI	*S.E.*	*P*-value
Abstract Reasoning	-0.064 (-0.123--0.005)	0.030	0.035
Numeric Memory	-0.068 (-0.124--0.012)	0.029	0.017
**Multiple falls**			
Abstract Reasoning	-0.126 (-0.173--0.078)	0.024	<0.001
Numeric Memory	-0.099 (-0.144--0.053)	0.023	<0.001

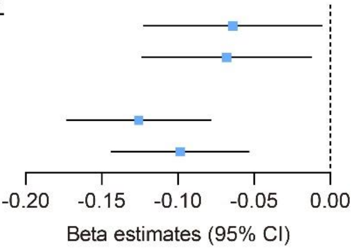

Supplementary Figure S5: Association between falls and cognitive decline in the UK Biobank

Both single and multiple falls were linked to decreased abstract reasoning (β range: –0.126 to –0.064, all $P < 0.05$) and numeric memory (β range: –0.099 to –0.068, all $P < 0.05$). Analysis adjusted for sex, age, education, Townsend deprivation index, alcohol consumption, smoking status, BMI, and the number of long-term morbidities. **UK: United Kingdom; CI, confidence interval; *S.E.*: standard error.**

Single falls	Participants with dementia, No./total No.	HR (95% CI)
Model 1: age and sex	4032/246428	1.28 (1.19-1.39)
Model 2: additionally included education and deprivation	4032/246428	1.27 (1.17-1.38)
Model 3: additionally included smoking and alcohol	4032/246428	1.27 (1.18-1.38)
Model 4: additionally included BMI	4032/246428	1.28 (1.18-1.38)
Model 5: additionally included long-term morbidities	4032/246428	1.24 (1.15-1.34)
Model 6: additionally included dementia polygenic risk score	4032/246428	1.23 (1.14-1.33)
**Multiple falls**		
Model 1: age and sex	3732/225383	1.98 (1.80-2.18)
Model 2: additionally included education and deprivation	3732/225383	1.92 (1.75-2.12)
Model 3: additionally included smoking and alcohol	3732/225383	1.87 (1.70-2.05)
Model 4: additionally included BMI	3732/225383	1.88 (1.71-2.07)
Model 5: additionally included long-term morbidities	3732/225383	1.70 (1.54-1.87)
Model 6: additionally included dementia polygenic risk score	3732/225383	1.66 (1.51-1.83)

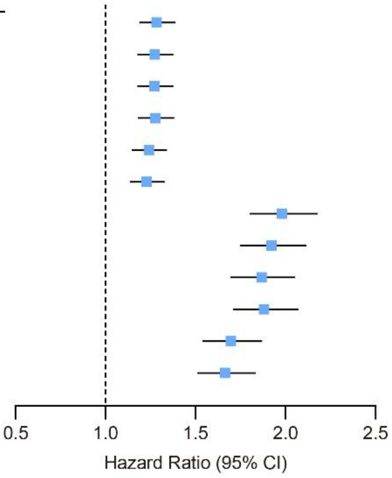

Supplementary Figure S6: Association between falls and the risk of dementia excluding movement disorders in the UK Biobank

After excluding participants with movement disorders, both single and multiple falls at baseline were associated with increased dementia risk after full adjustment (HR = 1.23 and 1.66, respectively; all *P* < 0.001). Model 1, adjusted for age and sex. Model 2, included model 1 plus education and Townsend deprivation index. Model 3, included model 2 plus smoking status and alcohol intake. Model 4, included model 3 plus body mass index. Model 5, included model 4 plus long-term morbidities. Model 6, included model 5 plus dementia polygenic risk score. UK: United Kingdom; CI: confidence interval; HR: hazard ratio; BMI: body mass index.

Supplementary Table S1: General characteristics of participants at baseline in the CHARLS

Characteristics	Falls at baseline		Incident dementia	
	**Non-fall (*N* = 4596)**	**Falls (*N* = 910)**	**No (*N* = 4693)**	**Yes (*N* =813)**
Age, years, mean (SD)	65.7 (6.1)	66.2 (6.4)	65.6 (6.1)	67.0 (6.5)
Sex, *N* (%)				
Female	2510 (54.6)	418 (45.9)	2518 (53.7)	410 (50.4)
Male	2086 (45.4)	492 (54.1)	2175 (46.3)	403 (49.6)
Education, *N* (%)				
Less than high school	4207 (91.5)	866 (95.2)	4306 (91.8)	767 (94.3)
High school or equivalent	291 (6.3)	31 (3.4)	287 (6.1)	35 (4.3)
College and higher	98 (2.1)	13 (1.4)	100 (2.1)	11 (1.4)
Household income, mean (SD)	25,559.3 (28808.8)	22,250.7 (23770.8)	25,936.6 (28805.3)	19,678.1 (22613.6)
Smoking status (yes), *N* (%)	2550 (55.5)	539 (60.9)	2626 (60.0)	463 (56.9)
Drinking status (yes), *N* (%)	2685 (58.4)	510 (55.6)	2735 (58.3)	460 (56.6)
BMI, mean (SD)	23.2 (3.7)	23.3 (4.0)	23.2 (3.7)	23.3 (4.0)
No. of long-term morbidities, *N* (%)				
None	1409 (30.7)	167 (18.4)	1422 (30.3)	154 (18.9)
1	1385 (30.1)	256 (28.1)	1431 (30.5)	210 (25.8)
2	955 (20.8)	220 (24.2)	973 (20.7)	202 (24.8)
3	474 (10.3)	151 (16.6)	501 (10.7)	124 (15.3)
4	231 (5.0)	69 (7.6)	235 (5.0)	65 (8.0)
⩾5	142 (3.1)	47 (5.1)	131 (2.8)	58 (7.1)

CHARLS: China Health and Retirement Longitudinal Study; BMI: body mass index; SD: standard deviation.

Supplementary Table S2: General characteristics of participants after propensity score matching in the Xuanwu cognitive cohort

**Characteristics**	**Total participants**			
	**Dementia (*N* = 474)**	**Control (*N* = 474)**	***P*-value**	**SMD**
Age, years, mean (SD)	66.9 (1.5)	66.9 (1.4)	0.787	0.030
Sex, *N* (%)				0.051
Female	220 (46.4)	232 (48.9)	0.474	
Male	254 (53.6)	242 (51.1)		
Education, *N* (%)				0.020
Less than high school	205 (43.2)	211 (44.5)		
High school or equivalent	227 (47.9)	221 (46.6)	0.598	
College and higher	42 (8.9)	42 (8.9)		
Smoking status (yes), *N* (%)	239 (50.4)	242 (51.1)	0.897	0.013
Drinking status (yes), *N* (%)	268 (56.5)	261 (55.1)	0.695	0.029
BMI, mean (SD)	27.3 (5.3)	27.3 (4.3)	0.922	-0.006
No. of long-term morbidities, N (%)				-0.029
None	132 (27.8)	128 (27.0)		
1	159 (33.5)	157 (33.1)		
2	104 (21.9)	106 (22.4)	0.938	
3	49 (10.3)	47 (9.9)		
4	19 (4.0)	26 (5.5)		
⩾5	11 (2.3)	10 (2.1)		
Falls				-
Non-fall, *N* (%)	343 (72.4)	385 (81.2)		
Single Falls, *N* (%)	83 (17.5)	56 (11.8)	0.005	
Multiple falls, *N* (%)	48 (10.1)	33 (7.0)		

BMI: body mass index; SD: standard deviation.

Supplementary Table S3: Association between falls and incident dementia in the CHARLS

Variable	Model 1		Model 2		Model 3	
	**HR (95% CI)**	***P*-value**	**HR (95% CI)**	***P*-value**	**HR (95% CI)**	***P*-value**
Non-fall	1 [Reference]	-	1 [Reference]	-	1 [Reference]	-
Falls	1.45 (1.25-1.69)	<0.001	1.41 (1.22-1.64)	<0.001	1.29 (1.11-1.50)	0.001

Model 1, adjusted for age and sex; Model 2, included model 1 plus education, household income, smoking status, drinking status, and BMI; Model 3, included model 2 plus long-term morbidities. CHARLS: China Health and Retirement Longitudinal Study; CI: confidence interval; HR: hazard ratio; BMI: body mass index.

Supplementary Table S4: Association between falls and incident dementia in the Xuanwu cognitive cohort

Variable	Model 1		Model 2		Model 3	
	OR (95% CI)	*P*-value	OR (95% CI)	*P*-value	OR (95% CI)	*P*-value
Non-fall	1 [Reference]	-	1 [Reference]	-	1 [Reference]	-
Single falls	1.71 (1.18-2.49)	0.005	1.72 (1.18-2.50)	0.004	1.72 (1.18-2.50)	0.004
Multiple falls	1.85 (1.15-2.97)	0.011	1.85 (1.15-2.97)	0.011	1.89 (1.17-3.02)	0.009

Model 1, adjusted for age and sex; Model 2, included model 1 plus education, smoking status, drinking status, and BMI; Model 3, included model 2 plus long-term morbidities. CI: confidence interval; OR: odds ratio; BMI: body mass index.

Supplementary Table S5: Association between falls and cognitive decline in the CHARLS

	**β**	**95% CI**	***P*-value**
Episodic memory	-0.029	-0.229, -0.027	0.013
Executive function	-0.041	-0.454, -0.141	<0.001

Analyses adjusted for sex, age, education, household income, smoking status, drinking status, BMI, and the number of long-term morbidities. CHARLS: China Health and Retirement Longitudinal Study; BMI: body mass index.

Supplementary Table S6: Association between brain structures and cognitive change in the UK Biobank

	β	*P*-value
**Abstract reasoning**		
Volume of grey matter (normalized for head size)	-0.004	0.670
Total volume of deep white matter hyperintensities	-0.033	0.002
Volume of grey matter in Hippocampus (left)	0.119	<0.001
Volume of grey matter in Hippocampus (right)	0.114	<0.001
Volume of grey matter in Parahippocampal Gyrus (left)	0.117	<0.001
Volume of grey matter in Parahippocampal Gyrus (right)	0.111	<0.001
**Numeric Memory**		
Volume of grey matter (normalized for head size)	0.019	0.395
Total volume of deep white matter hyperintensities	-0.035	<0.001
Volume of grey matter in Hippocampus (left)	0.078	<0.001
Volume of grey matter in Hippocampus (right)	0.063	0.002
Volume of grey matter in Parahippocampal Gyrus (left)	0.078	<0.001
Volume of grey matter in Parahippocampal Gyrus (right)	0.122	<0.001

Analyses adjusted for sex, age, education, Townsend deprivation index, alcohol consumption, smoking status, BMI, and the number of long-term morbidities. UK: United Kingdom; BMI: body mass index.

Supplementary Table S7: Association between PRS and incident dementia in the UK Biobank

	**Low PRS**	**Intermediate PRS**	**High PRS**
Cases/population	549/51,769	2450/155,979	2416/57,668
Hazard ratio (95%CI)	1 [reference]	1.50 (1.37-1.65)	4.12 (4.02-4.85)

UK: United Kingdom; CI: confidence interval; PRS: polygenic risk score.

Supplementary Table S8: Risk of incident dementia according to genetic and falls in the UK Biobank

Dementia PRS	Cases/population	Minimally adjusted HR (95% CI)	*P*-value	Fully adjusted HR (95% CI)	*P*-value	*P* value for interaction
**Low**						
non-fall	388/41,349	1 [Reference]		1 [Reference]		
single falls	95/7228	1.38 (1.10-1.73)	0.005	1.32 (1.05-1.65)	0.018	
multiple falls	66/3192	2.32 (1.78-3.01)	< 0.001	1.87 (1.43-2.45)	< 0.001	
**Intermediate**						
non-fall	1741/124,728	1 [Reference]		1 [Reference]		
single falls	414/21,855	1.32 (1.18-1.47)	< 0.001	1.26 (1.23-1.40)	< 0.001	0.001
multiple falls	294/9396	2.33 (2.06-2.64)	< 0.001	1.94 (1.71-2.21)	< 0.001	
**High**						
non-fall	1765/45,748	1 [Reference]		1 [Reference]		
single falls	414/8285	1.19 (1.07-1.32)	0.002	1.65 (1.05-1.30)	0.006	
multiple falls	237/3635	1.67 (1.46-1.92)	< 0.001	1.49 (1.29-1.71)	< 0.001	

Minimally adjusted: adjusted for age, and sex; Fully adjusted: adjusted for age, sex, education, Townsend deprivation index, alcohol consumption, smoking status, BMI, and the number of long-term morbidities. UK: United Kingdom; HR: hazard ratio; CI: confidence interval; BMI: body mass index.

Supplementary Table S9: Moderating effects of genetic risk on association between falls and brain structures in the UK Biobank

	**β**	**SE**	***P*-value**
Volume of grey matter (normalized for head size)	-118.421	673.669	0.861
Total volume of deep white matter hyperintensities	-65.358	53.073	0.218
Volume of grey matter in Hippocampus (left)	-5.873	7.148	0.411
Volume of grey matter in Hippocampus (right)	-3.453	7.373	0.640
Volume of grey matter in Parahippocampal Gyrus (left)	-16.793	8.958	0.061
Volume of grey matter in Parahippocampal Gyrus (right)	-11.926	9.234	0.197

Analyses adjusted for age, sex, education, Townsend deprivation index, alcohol consumption, smoking status, BMI, and the number of long-term morbidities. UK: United Kingdom; BMI: body mass index.

Supplementary Table S10: Association between falls and the risk of dementia stratified by subgroups in the UK Biobank

	**Non-falls HR (95%CI)**	**Single falls HR (95%CI)**	**Multiple falls HR (95%CI)**	***P*-value interaction**
**Age at enrolment**				
< 60 years old	1 [reference]	1.45 (1.23-1.67)	2.50 (1.89-3.31)	0.002
≥ 60 years old	1 [reference]	1.28 (1.19-1.38)	1.97 (1.80-2.16)	
**Sex**				
female	1 [reference]	1.39 (1.26-1.53)	2.13 (1.89-2.39)	0.315
male	1 [reference]	1.49 (1.34-1.66)	2.26 (1.99-2.58)	
**BMI**				
< 25	1 [reference]	1.35 (1.19-1.54)	1.89 (1.60-2.22)	0.414
≥ 25	1 [reference]	1.33 (1.22-1.45)	1.65 (1.49-1.84)	
**Ethnicity**				
White	1 [reference]	1.27 (1.17-1.37)	1.71 (1.56-1.88)	0.084
Others	1 [reference]	1.39 (1.07-1.79)	2.37 (1.80-3.15)	
**Education**				
Higher	1 [reference]	0.87 (0.57-1.34)	1.96 (1.30-2.97)	0.997
Others	1 [reference]	1.29 (1.21-1.40)	1.75 (1.60-1.92)	
**Smoking status**				
Never	1 [reference]	1.18 (1.06-1.32)	1.51 (1.33-1.72)	0.016
Previous/current	1 [reference]	1.37 (1.24-1.52)	2.06 (1.83-2.33)	
**Alcohol intake**				
Non-drinker	1 [reference]	1.26 (1.01-1.57)	1.78 (1.41-2.24)	0.659
Drinker	1 [reference]	1.28 (1.19-1.38)	1.76 (1.60-1.94)	

Note: Model adjusted for age, sex, education, Townsend deprivation index, alcohol consumption, smoking status, body mass index, and the number of long-term morbidities. Data are presented as HR (95% CI). UK: United Kingdom; HR: hazard ratio; CI: confidence interval.

Supplementary Table S11: Summary of cognitive functions assessed in the UK Biobank

Variable	UK Biobank Data Field ID	Definition
Abstract Reasoning (Fluid Intelligence)	20016	Fluid intelligence is assessed by giving participants 2 minutes to answer as many questions as possible, measuring their problem-solving skills using logic and reasoning, independent of prior knowledge.
Numeric Memory (Numeric Memory Test)	4282	The numeric memory test evaluates short-term memory for numbers. Participants initially see a 2-digit number, which disappears before they must recall and enter it. With each correct recall, the number length increases by one digit, up to 12 digits. Data collected include the longest sequence remembered and the time taken to complete the test.

UK: United Kingdom.

Supplementary Table S12: Definition and list of long-term morbidities

	Morbidity grouping*	Conditions included	Code
1	Hypertension	Hypertension	1065
		Essential hypertension	1072
2	Coronary heart disease	Heart attack/MI	1075
		Angina	1074
3	Diabetes	Diabetic nephropathy	1607
		Diabetic neuropathy/ulcers	1468
		Diabetes	1220
		Type 1 diabetes	1222
		Type 2 diabetes	1223
		Diabetic eye disease	1276
4	Stroke/TIA	Stroke	1081
		TIA	1082
		Subarachnoid haemorrhage	1086
		Brain haemorrhage	1491
		Ischaemic stroke	1583
5	Atrial fibrillation	Atrial fibrillation	1471
6	Heart failure	Cardiomyopathy	1079
		Hypertrophic cardiomyopathy	1588
		Heart failure/pulmonary oedema	1076
7	Peripheral vascular disease	Peripheral vascular disease	1067
		Leg claudication/intermittent claudication	1087
8	COPD	Chronic obstructive pulmonary disease/COPD	1112
		Emphysema/Chronic bronchitis	1113
		Emphysema	1472
9	Asthma	Asthma	1111
10	Bronchiectasis	Bronchiectasis	1114
11	Cancer*	“yes”/”no” to “have you ever had cancer?”	
12	Dyspepsia	Gastro-oesophageal reflux (GORD)	1138
		Oesophagitis/Barrett’s oesophagus	1139
		Gastric stomach ulcers	1142
		Gastric erosions/gastritis	1143
		Duodenal ulcer	1457
		Dyspepsia/indigestion	1510
		Hiatus hernia	1474
		Helicobacter pylori	1442
13	Diverticular disease	Diverticular disease/diverticulitis	1458
14	IrriSupplementary Table bowel syndrome	IrriSupplementary Table bowel syndrome	1154
15	Chronic liver disease	Oesophageal varices	1141
		Non infective hepatitis	1157
		Liver failure/cirrhosis	1158
		Primary biliary cirrhosis	1506
16	Inflammatory bowel disease	Inflammatory bowel disease	1461
		Crohn’s disease	1462
		Ulcerative colitis	1463

17	Constipation	Constipation	1599
		Hepatitis B	1579
18	Viral hepatitis	Hepatitis C	1580
		Hepatitis D	1581
19	Depression	Depression	1286
		Postnatal depression	1531
		Anxiety/panic attacks	1287
		Nervous breakdown	1288
		Post-traumatic stress disorder	1469
20	Anxiety	Obsessive compulsive disorder	1615
		Stress	1614
		Insomnia	1616
		Psychological/psychiatric problem	1243
	Schizophrenia/Bipolar affective disorder	Scizophrenia	1289
21		Mania	1291
	Bipolar	Bipolar disorder	1291
		Manic depression	1291
		Myositis/myopathy	1322
		Systemic lupus erythematosus/SLE	1381
		Connective tissue disorder	1373
		Sjogren's syndrome.sicca syndrome	1382
		Dermatopolymyositis	1383
22	Connective tissue diseases	Scloeroderma/systemic sclerosis	1384
		Rheumatoid arthritis	1464
		Psoriatic arthropathy	1477
		Dermatomyositis	1480
		Polymyositis	1481
		Polymyalgia rheumatica	1377
		Back pain	1534
		Joint pain	1537
		Headaches (not migraine)	1436
		Sciatica	1476
		Plantarfasciitis	1540
		Carpal tunnel syndrome	1541
		Fibromyalgia	1542
		Arthritis	1538
		Shingles	1573
23	Painful conditions	Disc problem	1532
		Prolapsed disc/slipped disc	1312
		Spine arthritis/spondylitis	1311
		Ankylosing spondylitis	1313
		Back problem	1294
		Osteoarthritis	1465
		Gout	1466
		Cervical spondylosis	1478
		Trigeminal neuralgia	1523

		Disc degeneration	1533
		Trapped nerve/compressed nerve	1257
24	Osteoporosis	Osteoporosis	1309
25	Thyroid disorders	Thyroid problem (not cancer	1224
		Hyperthroidism/thyrotoxicosis	1225
		Hypothyroidism/myxoedema	1226
		Grave’s disease	1522
		Thyroid goitre	1610
		Thyroititis	1428
26	Alcohol problems	Alcohol dependency	1408
		Alcoholic liver disease/alcoholic cirrhosis	1604
27	Chronic kidney disease	Polycystic kidney	1427
		Diabetic nephropathy	1607
		Renal/kidney failure	1192
		Renal failure requiring dialysis	1193
		Renal failure not requiring dialysis	1194
		Kidney nephropathy	1519
		Immunoglobulin A (IgA) nephropathy	1520
28	Prostate disorders	Prostate problem (not cancer)	1207
		Enlarged prostate	1396
		Benign prostatic hypertrophy	1516
29	Glaucoma	Glaucoma	1277
30	Epilepsy	Epilepsy	1264
31	Dementia	Dementia/Alzheimer/cognitive impairment	1263
32	Psoriasis or eczema	Eczema/dermatitis	1452
		Psoriasis	1453
33	Migraine	Migraine	1265
34	Chronic sinusitis	Chronic sinusitis	1416
35	Anorexia or bulimia	Anorexia, bulimia/other eating disorder	1470
36	Parkinson’s disease	Parkinson’s disease	1262
37	Multiple sclerosis	Multiple sclerosis	1261
38	Chronic fatigue syndrome	Chronic fatigue syndrome	1482
39	Endometriosis	Endometriosis	1402
40	Meniere disease	Meniere disease	1421
41	Pernicious anaemia	Pernicious anaemia	1331
42	Polycystic ovaries	Polycystic ovaries	1350

*Self-reported doctor-diagnosed conditions were obtained through nurse-led interviews (the UK Biobank data field ID: 20002), except for cancer, which was reported *via* touch-screen questionnaire. Disease groupings followed Barnett *et al* (2012)[1] classification. Patients with dementia (code 1263) were excluded from the baseline analysis. TIA, transient ischemic attack.

Case definition of dementia in relation to DSM-IV criteria

(Note this part reproduced from Ahmadi-Abhari et al., 2017 supplementary material[2])

We adapted the dementia case definition to resemble Diagnostic and Statistical Manual of Mental Disorders- IV (DSM-IV) and other criteria (such as National Institute of Neurological Disorders and Stroke-Association Internationale pour la Recherche et l'Enseignement en Neurosciences [NINDES-AIREN] and National Institute of Neurological and Communicative Disorders and Stroke-Alzheimer's Disease and Related Disorders Association [NINCDS-ADRDA]) for diagnosis of dementia. The cornerstone of clinical diagnostic criteria for dementia is impairments in two or more cognitive domains that result in considerable loss of function. Thus, we defined cognitive impairment as a score of equal to or lower than 1.5 standard deviations below mean, standardized to the population aged 50-80 with the same level of education, similar to criteria used for defining cognitive impairment no dementia (CIND).[3] Loss of function was defined as impairments in conducting activities of daily living. We sought with a set of criteria to encompass all types of dementia and not merely Alzheimer's disease. Although memory impairment is a key element in the diagnosis of Alzheimer's disease, memory is affected to varying degrees in vascular, fronto-temporal and Lewy body types of dementia. Thus, memory impairment was not included as a necessary criterion in defining cognitive impairment in this study.

DSM-IV criteria specify that the disturbances do not occur exclusively during the course of delirium and are not better accounted for by another mental disorder. For the criteria to hold, and to increase specificity, transient impairments in cognitive function or conducting Activities of Daily Livings (ADLs), were not classified as cognitive or functional impairment. Inclusion of impairments in conducting instrumental activities of daily living in case definition of dementia would have increased the sensitivity of our case-definition and would have enabled us to identify mild cases of dementia as well as the moderate to severe cases. However, this would also result in a great number of false positives. To ensure high specificity and to obtain unbiased estimates, we applied stringent criteria, requiring severe cognitive and functional impairment, for classification as dementia. As a result, only moderate to severe dementia cases are included in this study.

Reference

1. Barnett K, Mercer SW, Norbury M, Watt G, Wyke S, Guthrie B. Epidemiology of multimorbidity and implications for health care, research, and medical education: a cross-

sectional study. Lancet 2012; 380: 37-43.
2. Ahmadi-Abhari S, Guzman-Castillo M, Bandosz P, Shipley MJ, Muniz-Terrera G, Singh-Manoux A, et al. Temporal trend in dementia incidence since 2002 and projections for prevalence in England and Wales to 2040: modelling study. *Bmj* 2017;358:j2856.
3. Chertkow H, Nasreddine Z, Joanette Y, Drolet V, Kirk J, Massoud F, et al. Mild cognitive impairment and cognitive impairment, no dementia: Part A, concept and diagnosis. *Alzheimers Dement* 2007;3:266-282.
